# Utilization of Tires Waste-Derived Magnetic-Activated Carbon for the Removal of Hexavalent Chromium from Wastewater

**DOI:** 10.3390/ma14010034

**Published:** 2020-12-23

**Authors:** Waqas Ahmad, Shanif Qaiser, Rahman Ullah, Badrul Mohamed Jan, Michael A. Karakassides, Constantinos E. Salmas, George Kenanakis, Rabia Ikram

**Affiliations:** 1 Institute of Chemical Sciences, University of Peshawar, Peshawar 25120, Pakistan; waqasahmad@uop.edu.pk (W.A.); shanifqaisar888@gmail.com (S.Q.); rahmandawar@uop.edu.pk (R.U.); 2 Department of Chemical Engineering, University of Malaya, Kuala Lumpur 50603, Malaysia; badrules@um.edu.my; 3 Department of Materials Science and Engineering, University of Ioannina, GR-45110 Ioannina, Greece; mkarakas@uoi.gr (M.A.K.); ksalmas@uoi.gr (C.E.S.); 4 Institute of Electronic Structure and Laser, Foundation for Research and Technology-Hellas, N. Plastira 100, Vasilika Vouton, GR-70013 Heraklion, Crete, Greece; gkenanak@iesl.forth.gr

**Keywords:** tire waste, hexavalent chromium, magnetic activated carbon, wastewater treatment, tannery wastewater

## Abstract

The present study focuses on fabrication of magnetic activated carbon (M-AC) using tire waste and its potential investigation for adsorption of Cr (VI) from wastewater. The composite material (M-AC) was synthesized by pyrolysis followed by in situ magnetization method, and characterized by FTIR, FESEM, EDX, and XRD analysis. The maximum adsorption of Cr (VI) ion over composite adsorbent was found (~99.5%) to occur at pH 2, sample volume 10 mL, adsorbent dose 100 mg, contact time 30 min. The adsorption process was endothermic, feasible, spontaneous, and was found to follow pseudo second order of the reaction. The Cr ion could be completely desorbed (~99.3%) from the composite adsorbent by using 20 mL of 2 M NaOH solution. The composite adsorbent was regenerated by continuous adsorption and desorption for 5 consecutive cycles by using 10 mL 0.1 M HCl solution. M-AC also performed well in case of tannery wastewater by removing about 97% of Cr (VI).

## 1. Introduction

The spontaneous growth in global population as well as the advancement of industrial processes have been considered as the main source for the large accumulation of toxic metals in various water bodies [1]. In the past several years, large quantities of organic wastes such as pharmaceutical, food and beverages, textiles, pulp and paper, etc., from various industrial processes have been detected in tannery wastewaters [2,3]. Numerous methods such as precipitation, coagulation, filtration, bio-sorption, ion-exchange, adsorption, and stabilization have been utilized for Cr removal from wastewater [4,5,6,7,8]. Adsorption is currently the most widely used technique for the removal of heavy metals in a wide variety of wastewater matrices [9]. Nevertheless, a challenge therein is the development of an enhanced, cheap, and recoverable adsorbent that can effectively remove all types of the organic pollutants.

Environmentally, chromium (Cr) is one of the most widespread and threatening contaminants in wastewater [10]. Among significant heavy metals, Cr imparts a major role in water pollution as well as contamination of various nutritional plants [11]. The removal of Cr from wastewater has grasped the attention of the scientific community around the globe. To improve the environmental degradation, the presence of toxic Cr ions needs to be properly managed. The toxic effects of hexavalent chromium (Cr (VI)) have been observed through tanning, industrial dyes, mining, production of metals and alloys, corrosive paints, and electroplating [12,13]. As a dominant carcinogenic agent, Cr has caused hazardous effects through various cancerous diseases [14]. Adsorption has proven to be one of the promising methods to separate Cr (VI) from wastewater. However, selection of reliable adsorbents has made it crucial.

Researchers have shown reduction of Cr (VI) to Cr (III) using precipitation and coagulation [15]. Recent studies have reported novel mechanisms such as in situ deposition technique for the removal of heavy metals. The process involved deposition of electrodes to reduce Cr (VI) to Cr (III) inside the strip phase by incorporating iron and graphite rods as anodes [16,17].

Currently, an extensive research has been focused on the conversion of activated carbon (AC) from industrial wastes. This has led to the promotion of eco-friendly and cost-effective effects to commercialize AC. Natural, polymeric, zeolite, and nano adsorbents are reported as efficient and commonly used as remediation of wastewater treatment [18,19]. The conversion of tire waste into carbonaceous materials is an excellent alternative to environmental pollution, as it is believed to have the ability to enhance the absorption of organic pollutants and improve the removal efficiency. Until now, a lot of studies concerning the dispersion of magnetic iron oxide nanoparticles into carbon porous matrices have been reported [20,21,22,23]. However, the preparation and utilization of AC and magnetic AC (M-AC) from tire waste for Cr removal has rarely been reported in the literature [24].

In the current study, M-AC was synthesized from tire waste by acid demineralization and thermal pyrolysis at 450 °C followed by in situ magnetization. The prepared M-AC was characterized by FTIR, FESEM, EDX, and XRD analysis, and utilized as adsorbent for removal and recovery of Cr (VI) from tannery wastewater through batch mode adsorption. The adsorption parameters were optimized, and thermodynamics of adsorption was also investigated.

## 2. Experimental Work

### 2.1. Chemicals and Reagents

All chemicals used in this study were of analytical grade. Hydrochloric acid (HCl), Sulphuric acid (H_2_SO_4_) and Nitric acid (HNO_3_), and anhydrous Potassium dichromate (K_2_Cr_2_O_7_) were purchased from Merck KGaA, Darmstadt, Germany. Acetone (CH_3_COCH_3_), Sodium hydroxide (NaOH), and Methanol (CH_3_OH) were purchased from Sigma-Aldrich, St. Louis, MO, USA.

### 2.2. Sample and Materials Collection

Tanning wastewater containing Cr concentration 1640 mg/L was collected from Prime leather industries Pvt Ltd. (Sheikhopura, Punjab, Pakistan). The collected wastewater was stored in a plastic bottle, prewashed with diluted nitric acid, and immediately sent to the laboratory for determination of Cr concentration, removal, and recovery experiments.

To prepare activated carbon from the material, tire waste was collected from the local market (puncher shop) from Hayatabad Peshawar, Pakistan. The collected sample was washed with water several times to remove dust particles, and then dried in the oven at 60 °C for the whole night.

### 2.3. Preparation of Model Cr Solution

For the preparation of model Cr solution, 0.25 g of potassium dichromate (Sigma-Aldrich, St. Louis, MO, USA) was dissolved in 500 mL of distilled water, to obtain a solution containing 500 ppm Cr (0.0016 mol/L).

### 2.4. Preparation of the Adsorbent

M-AC was used for the adsorption and recovery of Cr (VI) ions. The stepwise synthesis and preparation of the adsorbent is given in details as below.

#### 2.4.1. Preparation of Activated Carbon

The collected tire waste was chopped with a paper cutter. For demineralization, the chopped tire waste (20 g) pieces were taken in a 100 mL beaker, and 30 mL of diluted nitric acid was added to it and allowed to digest for a few hours. After demineralization, the solid was separated from the mother liquid through filtration. The sample was washed with deionized water until it become neutral. The residue was dried in the oven at 60 °C.

The dried sample was heated in the tube furnace for pyrolysis. The tube used for the pyrolysis in the tube furnace was 14.7 cm in length and 2.54 cm in diameter. The tube was loaded from the sample, fitted in the tube furnace, and connected with inert gas (N_2_). The sample was heated for 4 h at 450 °C. After heating, the final sample was collected and stored for further study.

For activation, the sample (1 g) was ultrasonically dispersed in 100 mL conical flask. After the complete dispersion, 0.2 M potassium hydroxide (KOH; Sigma-Aldrich, St. Louis, MO, USA) (30 mL) was added and stirred for 3 h. The sample was separated from the liquid through Wattman filter paper, washed with distilled water till the washing was neutral, and then dried in the oven for 3 h at 60 °C.

#### 2.4.2. Magnetization of Activated Carbon

M-AC was prepared by direct method as reported in the literature [25]. Activated carbon (AC) 1.0 g was ultrasonically dispersed in deionized water for two hours. About 3.0 g FeCl_3_ (Sigma-Aldrich, St. Louis, MO, USA) and 5.5 g FeSO_4_ (Sigma-Aldrich, St. Louis, MO, USA) were added to the dispersion under continuous stirring, followed by addition of 10 mL of NH_4_OH (Sigma-Aldrich, St. Louis, MO, USA). A black precipitate was formed, which was separated from the suspension using an external magnet. The M-AC was excessively washed with distilled water and ethanol, and then dried under vacuum at 60 °C.

### 2.5. Characterization of Adsorbent

The tire waste-derived AC and M-AC was characterized by different instrumental analyses including FTIR, SEM, EDX, and XRD analysis. The FTIR analysis of the M-AC was carried out using an ATR-FTIR spectrophotometer (FTIR, PerkinElmer Spectrum 400, Amherst. MA, USA) equipped with an Attenuated Total Reflectance (ATR) diamond crystal plate and a pressure clamp; the spectra were collected at a resolution of 2 cm^−1^ and as the average of 50 scans over the spectral region 4000–500 cm^−1^. Surface morphology was examined through scanning electronic microscopy (SEM JSM 5910, JEOL, Japan). EDX analysis of the adsorbent was carried out by an EDX detector equipped with a SEM microscope. Structural parameters of the adsorbent were studied by a XRD analysis model (XPert^3^ Powder-Malvern Panalytical, Holland).

### 2.6. Batch Adsorption Experiments

For batch mode adsorption experiments, model Cr solution containing 500 mg/L (0.0016 mol/L) of Cr (VI) was prepared. About 10 mL of model Cr solution was taken in a conical flask, and the pH of the sample was set to 2 by using 1 M of HCl and NaOH solutions. About 100 mg of AC or M-AC was added to the sample and agitated on a flask shaker for 40 min. The adsorbent was separated from the sample with the help of an external magnet, and the sample was subjected to analyze Cr concentration. To achieve optimum conditions for adsorption, various parameters were optimized such as pH, adsorbent dose, contact time, temperature, etc., to calculate maximum adsorption.

### 2.7. Recovery of Cr from Adsorbent

The spent M-AC adsorbent was added to 20 mL of 2 M sodium hydroxide (NaOH) and agitated for 40 min on the flask shaker. The magnetic adsorbent was collected from the solution with the help of an external magnet, and the solution was analyzed for determining the concentration of Cr. The M-AC was then washed with deionized water, followed by diluted HCl, and dried at 60 °C, and the regenerated M-AC was ready for reuse in the next batch.

### 2.8. Analysis of Cr in Water Sample

The concentration of Cr was analyzed in waster sample through UV spectrophotometer (Shimadzu A90 UV-visible spectrophotometer; Shimadzu, Kyoto, Japan) using DPC method [26]. Diphenyl carbazide (DPC; Sigma-Aldrich, St. Louis, MO, USA) solution was prepared by dissolving 0.25 g DPC in 50 mL of acetone, which is used for complexation of Cr ions in this method. Standard solutions with different concentrations of Cr, i.e., 50, 75, 100, 150, 200, and 250 ppm were prepared for construction of calibration curve. About 10 mL of the standard solution was taken in a 50 mL beaker, and 1 mL of DPC solution was added to the standard solution, and its absorbance was determined at 543 nm. Likewise, the absorbance of the sample was also recorded, and the concentration of the Cr was determined from the calibration curve.

### 2.9. Calculations

The amount of Cr (VI) removed during the adsorption process was represented as % adsorption, which was calculated from the initial Cr concentration in sample (C_i_), and concentration after adsorption (C_f_) in mg/L, using the following relation.

(1) $$\%\;\mathrm{Absorption}=\;\frac{C_{i}-{\;C}_{f}}{C_{i}}\;\times 100$$

Similarly, the adsorption capacity at equilibrium (q_e_) was calculated using values of C_i_, C_f_, volume of sample (V), and mass of M-AC (m) through the following relation.

(2) $$\mathrm{qe}=\;\frac{(C_{i}-C_{f})\;\times V\;}{m}$$

Most commonly, the pseudo 1st and pseudo 2nd order kinetic models and intra-particle diffusion models are applied in the following mathematical forms [27,28].

(3) $$\frac{{\mathrm{dq}}_{t\;}}{\mathrm{dt}}\;=K_{1}\left(q_{e}-q_{t}\right)\;$$

(4) $$\frac{t}{q_{t}}=\;\frac{t}{q_{e}}\;+\;\frac{1}{k_{2}{\;q}_{e}{}^{2}}\;$$

(5) $$q_{t}=K_{1}t^{1/2}+\;C$$

In these equations, q_t_ is the adsorption capacity at any time “t” in mg·g^−1^, and q_e_ is the adsorption capacity at equilibrium in mg·g ^−1^. Whereas, *k_1_* (h^−1^) is the pseudo 1st order rate constant, which is determined from the slope of the plot of “ln (q_e_ − q_t_)” vs. time “t”. Similarly, *k_2_* (g·mg^−1^·h^−1^) is the pseudo 2nd order rate constant obtained from the slope of the plot between “t/q_t_” and “t”, whereas the value of “q_e_” is determined from intercept of the plot. Likewise, in Equation (5), q_t_ shows the intra-particle diffusion model, Ki is the intra-particle diffusion rate constant (g/mg min), and c is the intercept of the plot, which reproduces the boundary layer effect for the adsorption.

The following relations were used for calculation of ΔG°, ΔH°, and ΔS° [29].

∆G° = −RT ln K_D_ (6)

(7) $$∆H{}^{\circ}\;=\;R\;\frac{T_{2}T_{1}}{\;T_{2}-T_{1}}\ln\;\frac{K_{2}}{K_{1}}$$

(8) $$∆S{}^{\circ}\;=\;\frac{∆H{}^{\circ}\;-\;∆G{}^{\circ}\;}{T}$$

The following relation was used to calculate Langmuir adsorption isotherm [30].

(9) $$\frac{C_{e}}{q_{e}}\;=\;\frac{1}{q_{m}k_{b}}\;+\;\frac{1}{q_{m}C_{e}}\;$$

where, C_e_ is the final concentration of Cr in the wastewater in (mg/L), and q_e_ is the amount of Cr ions adsorbed on the adsorbent in (mg/g). Likewise, q_m_ is the maximum adsorption limit, and K_b_ is Langmuir constant, which is related to energy. Another very important parameter, called dimensionless isolating component (R_L_), is calculated from Langmuir isotherm by applying the following expression [31].

(10) $$R_{L}=\;\frac{1}{1\;+k_{b}C_{e}}\;$$

To study Freundlich adsorption isotherm, the following relation was used [32].

(11) $${\mathrm{logq}}_{e}=\;\mathrm{logKf}\;+\;\frac{1}{n}{\mathrm{logC}}_{e}$$

## 3. Results and Discussion

In the current research, the adsorption and recovery of Cr-VI from wastewater was studied using M-AC as adsorbent. The M-AC was prepared from tire waste, and then characterized by various instrumental analysis. The adsorption of Cr-VI from the model and wastewater was studied in batch mode experiments.

### 3.1. Characterization of Adsorbent

#### 3.1.1. FTIR Analysis

**Figure 1:**
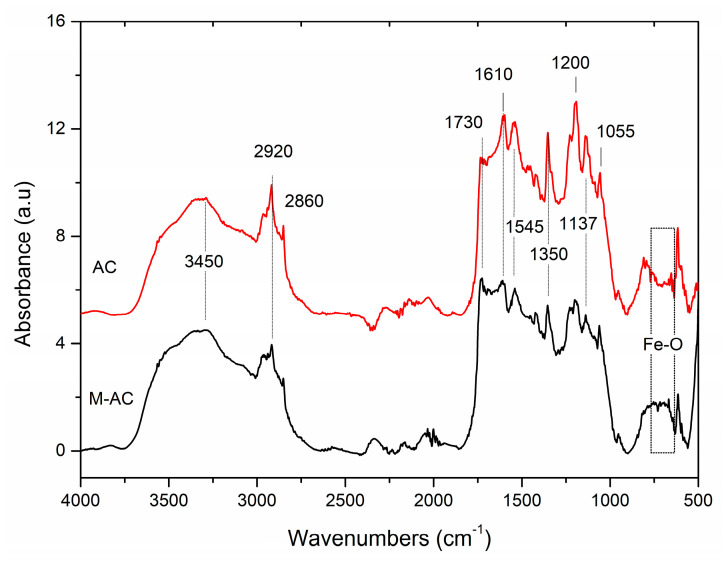
FTIR spectra of tire waste-derived activated carbon (AC) and magnetic AC (M-AC).

Figure 1 presents the FTIR spectra of the AC (a) and M-AC (b). The infrared spectrum of AC exhibits two maxima at around 1610 cm^−1^ and 1730 cm^−1^. The first one can be assigned to the stretching vibrations of C=C bonds in aromatic carbon rings and pyrone structures as also the band at ~1545 cm^−1^. The second absorption band at 1610 cm^−1^ can be assigned to asymmetric stretching vibrations of -COOH carboxyl and -COO^−^ carbonyl, and/or -C=O ketone units. The band at 1350 cm^−1^ can be attributed to symmetric stretching vibrations of COOH groups, whereas the bands around 1200 cm^−1^ to asymmetric stretch of -C-C-C bridges in ketonic groups and/or to deformation vibrations of O-H in the carboxylic acid groups. Peaks positioned at 1137 cm^−1^ and 1055 cm^−1^ show C-O ether and Si-O moieties [33]. The band centered at 3450 cm^−1^ is assigned to the O-H stretching modes of the -COOH, and phenolic OH groups; the weak bands at 2920 and 2860 cm^−1^ are assigned to CH_3_ and CH_2_ stretching vibration modes. The FTIR spectrum of M-AC shows similar configurations as that of AC, however, an additional absorption appears between 620–750 cm^−1^, which attributes to Fe-O stretching vibrations of magnetic iron oxides [34].

#### 3.1.2. XRD Analysis

**Figure 2:**
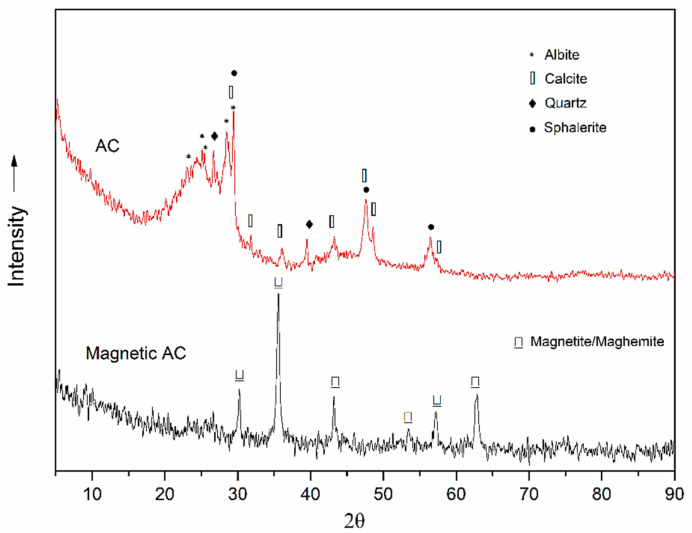
XRD patterns of tire waste-derived AC and M-AC.

In the case of AC, the diffractogram (Figure 2) exhibits two characteristic broad reflection peaks centered at 2θ, 25°, and 43°, which can be attributed to the amorphous nature of activated carbon. On this background appear several sharp reflection peaks at 2θ 26.7, 28.5, 29.7 31.8, 36, 39, 7.6, and 56.5°. These peaks may be assigned to quartz SiO_2_ (main peak at 26°; OCD: 96-900-0776) [20], calcite (main peak at 29.7°; OCD: 96-101-0963), albite, NaAlSi_3_O_8_ (25.2, 26, 28.5 and 29.7°; OCD: 96-900-3703) and sphalerite, ZnS (28.5, 36, 47.6 and 56.5°; OCD: 96-900-0108), which are assumed to be remains of the filler materials, or formed after pyrolysis [21]. The diffractogram of M-AC shows peaks at 2θ of 30, 35.5, 53.6, 57.6, and 62.8°, which indicate the crystalline patterns of magnetite (Fe_3_O_4_; 96-900-2317) or maghemite (γ-Fe_2_O_3_; OCD: 96-900-6317) [22], which indicates that both the magnetic oxides of iron, i.e., magnetite and maghemite, are impregnated on the surface of the AC.

#### 3.1.3. FESEM Analysis

**Figure 3:**
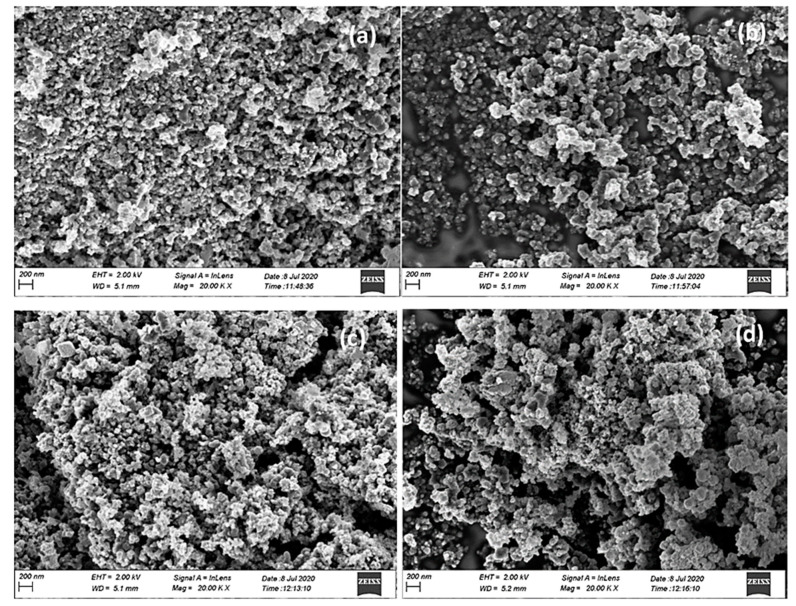
FE-SEM micrographs of tire waste-derived AC (**a**,**b**) and M-AC (**c**,**d**).

The morphology of the AC and M-AC was investigated by FESEM microscopy. The FESEM micrographs of the AC and M-AC are indicated in Figure 3a–d. The micrographs of AC, indicated in lower and higher magnification (Figure 3a,b), show the highly porous and granular morphology of AC. The granules are stacked in layers, as evident from the FESEM micrograph, and the grain size is estimated to be in micrometer-sized particles. Some surface granules seem agglomerated, but deep caves and fissures can be seen. The M-AC (Figure 3c,d), also represents a similar morphology, however, the granules seem covered with a spongy layer of magnetic oxides. Additionally, more agglomeration can be observed on the surface, though the fissures size has been increased. Appearance of tire waste-derived AC as spherical nanometric particles is in agreement with literature reports [35].

#### 3.1.4. EDX Analysis

**Figure 4:**
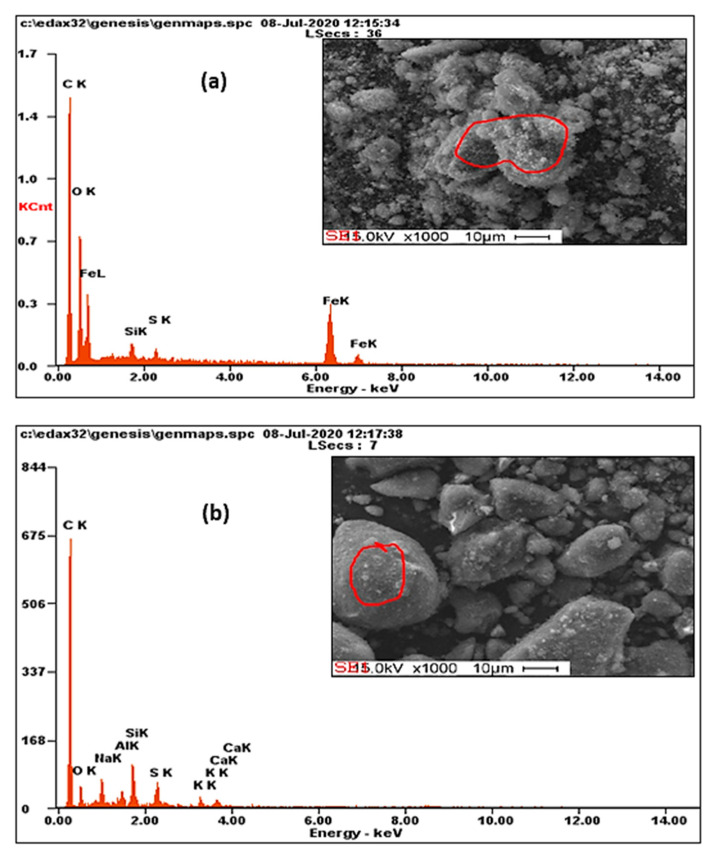
EDX profiles of tire waste-derived M-AC (**a**) and AC (**b**).

The elemental composition of the AC and M-AC was investigated by EDX analysis (Oxford Instruments, High Wycombe, United Kingdom). The EDX profiles of the samples are shown in Figure 4a,b, whereas the percentage of the various elements found in AC and M-AC is given in Table 1. In the case of AC, the percentage of C and O was found to be 76 and 8%, and the high quantity of C confirms the synthesis of activated carbon, whereas O may be present as surface functional groups of AC or as oxides. Additionally, some other elements, including Na (3%), Al (1%), Si (5%), S (3%), K (1%), and Ca (1%), which may be present as oxide impurities or as mineral clays which may be added to rubber as fillers, out of these sulfurs may be present in the tire waste in elemental form. Jha et. al. [36] has also reported the presence of various minerals, i.e., anorthite, spinel oxides, and quartz, etc., in the activated charcoal derived from tire waste char. In case of M-AC weight, percentages of C, O, and Fe were found to be 46, 21, and 30%, respectively, which confirm the incorporation of magnetic iron oxides on AC. The decrease in the concentration of other elements in the M-AC which were present in AC may be attributed to their extraction during magnetization and washing steps.

### 3.2. Adsorption Experiments and Effect of Process Parameters

The removal of Cr (VI) from model Cr solution was studied through batch mode adsorption using M-AC prepared from tire waste. The effects of various adsorption parameters on % adsorption of Cr were investigated to optimize the conditions for maximum removal of Cr from the model Cr solution.

**Figure 5:**
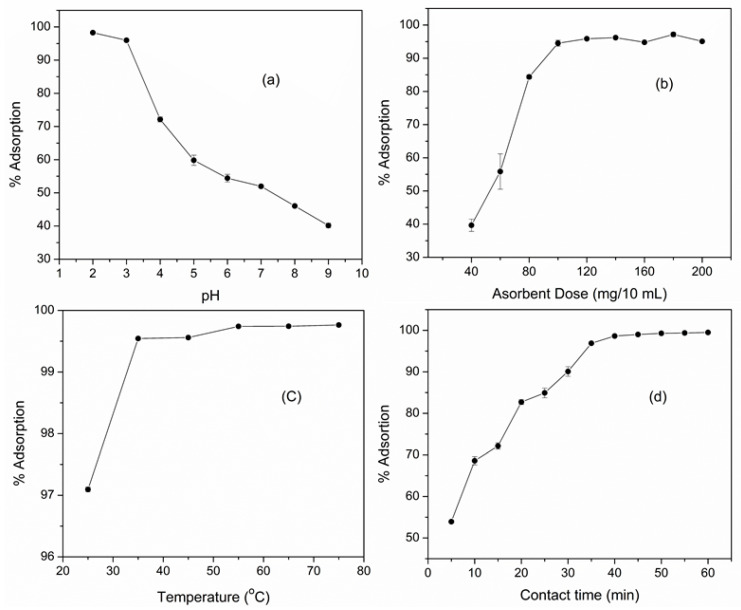
Effect of pH (**a**), adsorbent dose (**b**), temperature (**c**) and contact time (**d**) on the adsorption of Cr (VI) over M-AC.

Adsorption of Cr (VI) over M-AC was studied under a pH range of 2 to 9, results are displayed in Figure 5a, and the results show that maximum adsorption of about 98% occurs at low pH, i.e., pH 2, whereas with increasing the pH of the media, the % adsorption of Cr declines. Since at low pH the adsorbent surface remains protonated, hence the anionic Cr species are adsorbed on the surface through electrostatic interaction [37]. It has been shown that in an aqueous medium, Cr may exist in different ionic forms such as chromate (CrO_4_^−2^), dichromate (Cr_2_O_7_^−^), and hydrogen chromate (HCrO_4_^−^), which primarily depend upon the pH of the medium [38]. Generally, under acidic medium, the predominant oxy anionic Cr species are HCrO_4_^−^ and Cr_2_O_7_^−2^ ions, whereas under basic medium, the CrO_4_^−2^ is more stable [39]. As CrO_4_^−2^ ion carries two negative charges, hence it requires two cationic sites for adsorption on the surface of the adsorbent, whereas HCrO_4_^−^or Cr_2_O_7_^−^ ions carry one negative charge and therefore each need a single cationic site for adsorption [40], this means two fold number of HCrO_4_^−^ or Cr_2_O_7_^−^ ions can be adsorbed on the same number of cationic sites as compared to that of CrO4^−2^ ions. Thus, under acidic conditions, more Cr can be adsorbed over the adsorbent surface because in acidic medium, Cr predominantly exists as HCrO_4_^−^ or Cr_2_O_7_^−^ ions, while requiring single cationic site, a large number of these ions will be adsorbed on a smaller unit surface area of the adsorbent. Moreover, at low pH, competitive adsorption of OH^−1^ ions also declines the adsorption of Cr anionic species [41].

Adsorption efficiency of M-AC was also studied under different adsorbent doses ranging from 40 to 200 mg/10 mL, and results shown in Figure 5b indicate that adsorption of Cr (VI) increases with the increasing the adsorbent dose, and maximum adsorption of 94.5 ± 1 is attained by using a 100 mg/10 mL dose of M-AC, however, no further increase in adsorption occurs with the increasing of the adsorbent dose. Since a higher adsorbent dose offer more adsorption sites on the adsorbent surface, therefore the adsorption activity increased with the increasing adsorbent dose [42]. These results agree with literature reports, where different types of adsorbents have also been shown to attain maximum adsorption of Cr(VI) at an optimum dose of 100 mg [13,43].

The effect of temperature on adsorption of Cr (VI) between the temperature range of 25–75 °C has been shown in Figure 5c. The data reveals that the adsorption abruptly increases with increasing temperature up to 35 °C, beyond which the adsorption activity almost remains constant. This may be attributed to the increase in the diffusion rate of the metal ion with rise in temperature [44]. Similarly, the effect of contact time on the adsorption activity was studied, which indicated that the adsorption of Cr (VI) over M-AC increased with the increase in contact time (Figure 5d). Equilibrium is attained in 30 min, and with further increase in contact time, the adsorption activity stays constant.

It may be concluded from the above discussion that the optimum conditions for the maximum adsorption of Cr (VI) ions (99.5%) over M-AC are; pH 2, a sample volume of 10 mL, an adsorbent dose of 100 mg/10 mL, 35 °C temperature, and a contact time of 30 min.

### 3.3. Kinetic Study

The effect of temperature on the Cr (VI) adsorption over M-AC has been described above, which revealed that with increasing temperature, the adsorption rate increases, and equilibrium adsorption is establish at 35 °C, as further increasing in the temperature causes no increase in adsorption of Cr. A better description of adsorption rate can be explained by the interpretation of the adsorption data using various kinetics models.

**Figure 6:**
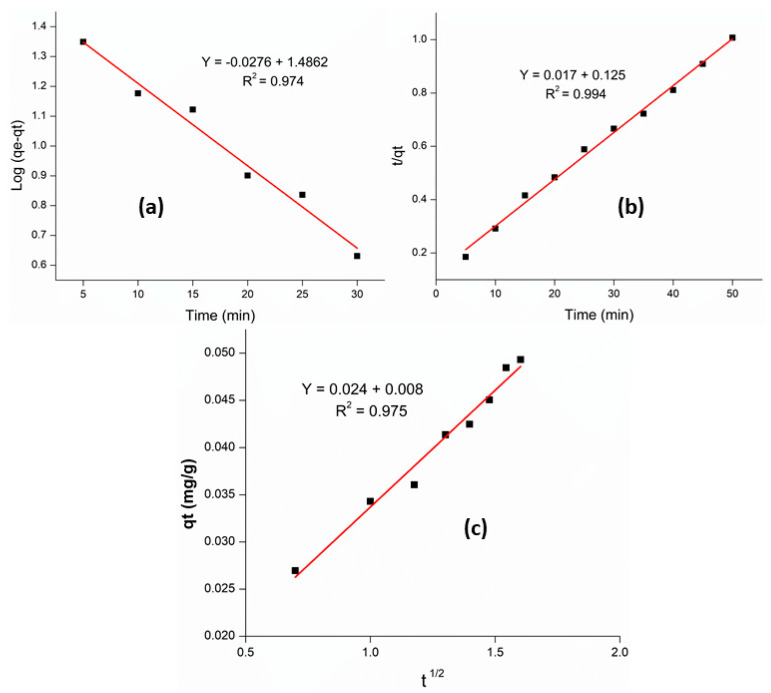
Pseudo 1st order (**a**), pseudo 2nd order (**b**), and intra particle diffusion (**c**) kinetic plot for adsorption of Cr (VI) over M-AC.

Table 2 shows the values of different kinetic parameters. It can be observed from the Figure 6a,b that the pseudo 2nd order model gives straight line with higher R^2^ value (0.99) than that of the pseudo 1st order kinetic model (0.97). Similarly, the experimental value of q_e_ has been found to be 49.314 mg·g^−1^ (Table 2), which is more close to “q_e_” calculated by the pseudo 2nd order model (58.82 mg·g^−1^) as compared to that of the pseudo 1st order kinetic model (30.63 mg·g^−1^). These results conclude that the adsorption of Cr (VI) over M-AC is in better agreement with the pseudo 2nd order kinetic model. Similarly, the intra-particle diffusion gives a linear plot with high R^2^ value (0.97), which means that the adsorption data agrees closely with the intra-particle diffusion model as shown in Figure 6c.

A number of research reports shows that Cr(VI) adsorption over different adsorbents such as M-AC derived from termite feces [45], acid treated saw dust, and saw dust carbon [46] follow pseudo 2nd order kinetics. Nevertheless, several other studies show that the adsorption of Cr(VI) over the adsorbents, including AC obtained from olive stones [47] and graphene oxide [48], is in accordance with pseudo first order kinetics. This shows that the adsorption kinetics of Cr (VI) depend on the nature of the adsorbent material.

### 3.4. Thermodynamic Studies

Certain thermodynamic parameters provide important information about the nature of the adsorption phenomena and energy changes involved in it. Various important thermodynamic parameters, including Gibbs free energy (ΔG°), enthalpy (ΔH°), and entropy (ΔS°) were calculated from the adsorption data.

The results are given in Table 3, which indicates that at different temperatures, the values of ΔG° are found to be negative, and also the negative values of ΔG° increase with increasing the temperature. These results indicate that the adsorption of Cr (VI) on M-AC is feasible and spontaneous in nature [49]. Additionally, the feasibility of adsorption increases with increases in temperature, and this is also in accordance with the effect of temperature on adsorption activity of the M-AC discussed in previous section. The value of enthalpy change (∆Hº) was found to have been calculated to be 26.09 KJ·mol^−^^1^, which indicates that the adsorption process is endothermic in nature. It has been reported in literature that the adsorption system, having an ∆Hº value between 2.1 to 20.9 KJ·mol^−^^1^, the interaction of the adsorbate on the adsorbent surface will predominate through electrostatic attraction, which means the adsorption is physisorption, while if the value of ∆Hº occurs in the range of 20.1 to 418.4 KJ·mol^−1^, then transfer of charges may be involved between the adsorbate and adsorbent, driving the adsorption through chemisorption [50]. Since in the current scenario, the value of ∆Hº is 26.09 KJ·mol^−^^1^, i.e., above 20.9 KJ·mol^−^^1^, hence the current adsorption system is chemisorption, which might involve the formation of a coordinate covalent bond between Cr(VI) ions and M-AC. Likewise, the value of change in entropy (∆Sº) is found to be 0.162 KJ·mol^−^^1^k^−^^1^, which indicates the adsorption process is spontaneous and governed by entropy rather than enthalpy [51].

### 3.5. Adsorption Isotherms

**Figure 7:**
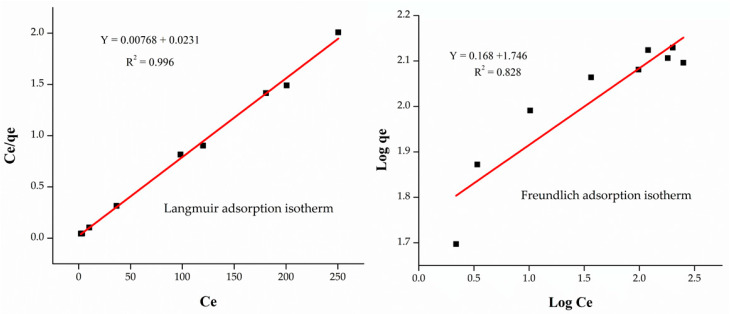
Plots of Langmuir and Freundlich adsorption isotherms for adsorption of Cr (VI) over M-AC.

To study the adsorption process, the experimental data was fitted into Langmuir and Freundlich isotherms. The adsorption process greatly depends on the R_L_ value. The R_L_ value in the result was found to be 0.352, which confirmed that the adsorption process is progressive and favorable. From the Langmuir isotherm graph, slope and intercept values were obtained, which verified the adsorption process may be favorable or unfavorable, reversible or irreversible, as shown in Table 4. The graph was plotted between C_e_/q_e_ and C_e_, as shown in Figure 7. The results indicated that the maximum adsorption capacity was found to 142.85 mg/g, coefficient correlation factor (R^2^) value was (0.996) achieved, while Langmuir constant value was (K_b_ = 0.303) found, respectively.

The Freundlich adsorption isotherm gives information about whether the adsorption process is heterogeneous or not. The slope and intercept values are achieved with the Freundlich adsorption isotherm graph, as presented in Figure 7. In the Freundlich isotherm, 1/n value achieved 0.168, which indicates that the adsorption process is applies over the whole range of concentrations [52].

The current result indicates that the R^2^ values for Langmuir and Freundlich adsorption isotherm were found to be (0.996) and (0.828), respectively, which means Langmuir adsorption isotherm fits more closely as compared to Freundlich. Likewise, the R_L_ value was found to be 0.352, which indicates the adsorption process is monolayer and favorable. Similarly, the 1/n value from the Freundlich isotherm was found to be 0.168, which showed that the adsorption process is favorable at all possible concentrations.

### 3.6. Recovery of Cr(VI) and Regeneration of Adsorbent

In any adsorption process, the regeneration of the adsorption is a crucial step, which has a major economic impact on the industrial scale application of the process. The economy of the adsorption process depends upon how many times an adsorbent can be reused without losing its adsorption efficiency [53]. The recovery of Cr (VI) from the M-AC was carried out through desorption by leaching with various alkaline solutions. During the desorption process, the Cr (VI) loaded M-AC was stirred with 1 M and 2 M solutions of NaOH and NH_4_OH, respectively. M-AC was recovered from the solution with the help of an external magnet, and the concentration of Cr (VI) in the alkaline solution was analyzed. Results given in Table 5 show that, NaOH solution was more effective than NH_4_OH in desorption of Cr (VI) from the surface of M-AC, and the highest Cr (VI) desorption of about 56% was attained by using 2 M NaOH. Desorption of Cr (VI) from the surface adsorbent using basic solutions was critically reviewed. Many literature reports have also confirmed the high efficiency of NaOH solution in desorption of Cr(VI) from the surface of a variety of adsorbents [54,55].

**Figure 8:**
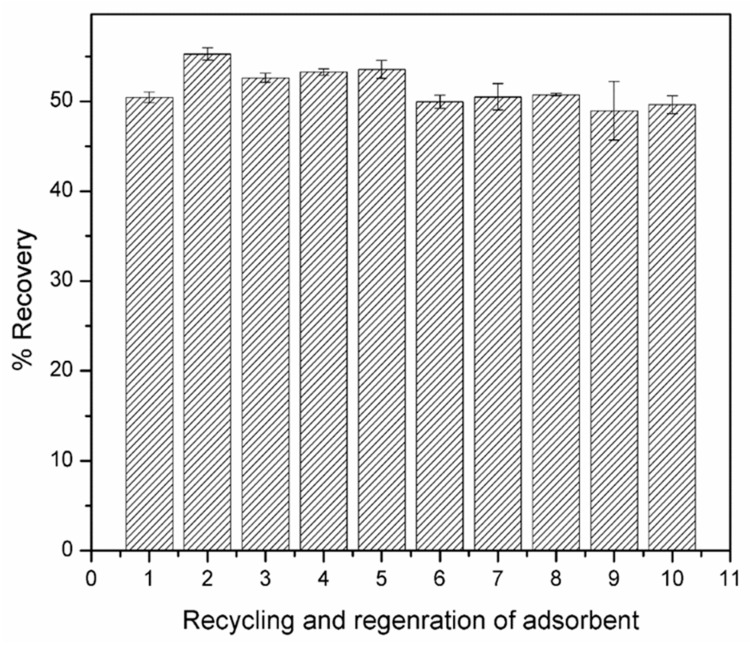
Recovery of Cr (VI) during repeated adsorption of desorption processes.

After desorption, the M-AC was regenerated by washing with HCl (0.1 M) and deionized water followed by vacuum drying at 60 °C. The regenerated M-AC was further used for adsorption experiments using a fresh batch of Cr (VI) solution. The adsorption and desorption processes were repeated for several cycles. Results indicated that the Cr (VI) adsorption efficiency of M-AC was almost the same (95%) for about ten repeated cycles. Additionally, the recovery or desorption efficiency of the M-AC also remained constant (about 51–55%) for five consecutive cycles (Figure 8). This shows that the current adsorption system is not only helpful in the removal of C(VI), but also helps in the recovery of industrially important Cr metal from industrial effluents, which may be recycled and reused in other applications.

### 3.7. Mechanism of Adsorption

The mechanism of adsorption in the present study was due the interaction between hydrogen chromate (HCrO_4_^−^) and the surface of M-AC. The main factor involved in the mechanism of adsorption of Cr (VI) is pH. The maximum adsorption of Cr (VI) was found at pH 2. At this pH, Cr_2_O_7_^−2^ is converted to HCrO_4_^−^ species, as shown in the reaction [56].

Cr_2_O_7_^−2^ + H_2_O → 2HCrO_4_

The adsorptive capacity of magnetic composite was due to the active sites, present in both organic phases and inorganic, as well as new sites yield from the interaction of both phases. The adsorption of HCrO_4_^−^ on active sites of organic and inorganic involves electrostatic interaction. The oxygen atom on the surface of the magnetic composite became protonated at pH 2 to a great extent, which resulted in a strong electrostatic attraction between HCrO_4_^−^ and positively charged adsorbent. At this condition, toxic Cr (VI) in tannery waste was adsorbed onto the surface of adsorbent (MNPs) by electrostatic attraction.

Studies have shown that iron oxide particles impart excellent adsorption properties due to their desirable surface characteristics [57]. In the present study, their impregnation on AC was intended for the ease of separation as adsorbent from the wastewater sample. Due to their low concentration over AC, their contribution towards adsorption has been considered very small. It is also probable that the electron donating surface functionalities of M-AC, as well as magnetic Fe^+2^, may cause the reduction of Cr (VI) to Cr (III), which may further be adsorbed over anionic site via ion exchange adsorption mechanism [58].

### 3.8. Treatment of Tannery Wastewater

Preliminary characterization of the tannery wastewater sample is presented in Table 6. Results show that the pH, concentration of Cr, chemical oxygen demand (COD), and suspended solids in tannery wastewater were 3.17, 1640 mg/L, 1130 mg/L, and 960 mg/L, respectively. According to Environmental Health Safety Guidelines (2009), the permissible limit for pH and COD in wastewater is 6–9 and 150 mg/L. Likewise, EPA guidelines limit the concentration of Cr in wastewater up to 200 ppm. These guidelines suggest that the tannery wastewater exceeds the permissible limits, especially in case of Cr concentration, therefore proper treatment is required to reduce the Cr concentration before disposal.

The adsorption of Cr from tannery wastewater was investigated over M-AC under optimized conditions. Results presented in Table 6 display that about 97% Cr was removed from tannery wastewater during adsorption. This shows that the magnetic AC (M-AC) also performed well in case of tannery wastewater like the model Cr solution. Hence, the adsorbent can be used on an industrial scale for treatment of tannery wastewater.

### 3.9. Comparison of Adsorption Efficiency

A variety of activated carbon-based adsorbents derived from low-cost carbonaceous waste materials have been used for adsorption of Cr (VI). These include various biomass materials and activated carbon derived from different biomass wastes such as olive stones, coconut shells, sugar wastes, pine leaves, saw dust, cactus leaves, etc. A summary of the comparative adsorption efficiencies of such adsorbents reported for removal of Cr (VI) ions with that of M-AC used in the current study has given in Table 7. It is clear from the comparison data that the M-AC offers high Cr (VI) adsorption efficiency under mild conditions and short contact time compare to the reported adsorbents.

## 4. Conclusions

A well organized and novel adsorbent magnetic activated carbon (MNPs-AC) composite was synthesized and used as an adsorbent for adsorption of Cr (VI) ions from tannery wastewater. The composite material was analyzed by FTIR, XRD, and SEM. The FTIR analysis confirmed the successful synthesis of M-AC. Whereas, SEM analysis shows surface morphology of (MNPs-AC) composite, indicating a rough surface, MNPs are uniformly distributed on the surface, which offer a large exposed surface area for the adsorption of metal ions. During the batch study, an external magnetic field was used to separate the adsorbent from sample solution. At optimum conditions, (99.32 ± 2%) adsorption of Cr (VI) ions was found at pH 2, adsorbent dose 100 mg, sample volume 10 mL, vortex time 30 min. The adsorption of Cr (VI) adsorbent composite was found to be pseudo second order and endothermic in nature. The adsorbent showed high efficiency for ten cycles of uses, after regeneration. The Cr (VI) ion was successfully desorbed (56 ± 1%) from the surface of adsorbent by using 20 mL of 2 M NaOH solution.

## Figures and Tables

**Table 1:** Elemental composition of tire waste-derived AC and M-AC.

Element	AC		Magnetic AC	
	Wt%	At%	Wt%	At%
C	75.98	85.53	45.81	66.05
O	08.63	07.30	21.43	23.20
Na	03.08	01.81	-	-
Al	01.41	00.71	-	-
Si	04.94	02.38	01.25	00.77
S	03.08	01.30	00.89	00.48
K	01.44	00.50	-	-
Ca	01.44	00.48	-	-
Fe	-	-	30.62	09.50

**Table 2:** Kinetic parameter of the adsorption of Cr (VI) on the surface of (MNPs-AC), pH 2, adsorbent dose 100 mg, and vortex time 35 min.

Experimental	Pseudo First Order			Pseudo Second Order			Intra-Particle Diffusion Model		
q_e_ (mg·g^−1^)	K_1_ (min^−1^)	q_e_ (mg·g^−1^)	R^2^	K_2 _ (min^−1^)	q_e_ (mg·g^−1^)	R^2^	K_i _ (g/mg.min)	C	R^2^
49.314	0.064	30.63	0.97	0.0023	58.82	0.99	0.024	0.008	0.97

**Table 3:** Thermodynamic parameter for the adsorption of Cr (VI) over M-AC.

Temperature (K)	ΔGᵒ (KJmol^−1^)	ΔHᵒ (KJmol^−1^)	ΔSᵒ (KJmol^−1^k^−1^)
298	−21.58	26.069	0.162
308	−24.54		
318	−25.43		
328	−27.68		
338	−28.56		
348	−29.92		

**Table 4:** Adsorption isotherm models, for adsorption Cr (VI) on M-AC composite.

Isotherm Model	R^2^	Kf	1/n	K_b_	q_m_ mg·g^−1^	R_L_
Langmuir Isotherm	0.996	-	-	0.303	142.85	-
Freundlich Isotherm	0.828	0.242	0.168	-	-	0.352

**Table 5:** Influence of various eluents on the desorption of Cr (VI) ions from the adsorbent.

Leaching Solution	Cr (VI) Recovery (%)
1 M NaOH	45 ± 2
2 M NaOH	56 ± 1
1 M NH_4_OH	30 ± 2
2 M NH4OH	35 ± 2

**Table 6:** Characteristics of tannery wastewater and removal of Cr.

Parametre	Values
pH	3.17
COD	1130 mg/L
Suspended solids	960 mg/L
Concentration of Cr	1640 mg/L
Removal of Cr through adsorption	97%

**Table 7:** Comparison of Cr (VI) adsorption efficiencies of various low-cost adsorbents.

Adsorbent Materials	Experimental Conditions	Adsorption Capacity	Reference
Acid activated Carbon derived from olive stones	pH 1.5, Cr conc. in water 4–50 mg/L, Adsorbent dose 0.3 g	71 mg/g	[47]
Coconut shell charcoal (CSC) and commercial activated carbon (CAC)	Cr conc. 5,10, 20, and 25 mg/L	CAC at 4.7 mg/g & CSC at 5 mg/L	[59]
Wool, sawdust, pine needles, almond shells, cactus leaves, and charcoal	Cr conc. 20, 100, 200, 300, 400, 500 and 1000 mg l^−1^, Adsorbent conc. 16 g l^−1^ at 30 °C	81% out of 100 ppm Cr(VI)	[60]
Green coconut shell	Cr conc. 10–100 mg/g, T range 10–80 °C	22.9 mg/g (90% for 10 mg/L)	[61]
Activated carbon (C1, C2, C3) from industrial sugar waste	pH 5–6, T 28 °C, Cr conc. 0.15 and 0.7 mg/L	C1 98.86, C2 98.6 and C3 93%	[62]
Activated carbon (1), calcinated egg shells (2), wheat bran (3), modified wheat bran (4)	Cr conc. 10 mg/L, rpm-180, T 35 °C	98.75% (1), 64% (2), 75.89% (3), 96.96% (4)	[63]
Silver impregnated groundnut husk (1), activated carbon from groundnut husk (2)	pH 1–3, Cr conc. 0.5 g/100 mL	11.4 mg/g (1), 7.0104 mg/g (2)	[64]
Inexpensive carbohydrates derived supramolecular gels	pH 7.4, 250 mg or 20 mg of gel at 5 wt% in 10 or 20 mL Cr solutions	598 mg/g (97% in 24 h)	[65]
Porous activated carbon from *Camellia oleifera* seed	pH 2–8, Cr conc. 30 mg L^−1^, T 25 °C	307.3 mg/g	[66]
Carbon nanocomposite from natural diatomite	pH 1, Cr conc. 50–300 mg/L, T 298.15 K	142.9 mg/g	[67]
Magnetic activated carbon (M-AC) from tires waste	pH 2, 35 °C, 40 min, 100 mg adsorbent	49.3 mg/g	Current study

## Data Availability

Data sharing not applicable, all the data created for this study is already displayed in the article.
